# Cognitive remediation improves cognition and good cognitive performance increases time to relapse – results of a 5 year catamnestic study in schizophrenia patients

**DOI:** 10.1186/1471-244X-13-184

**Published:** 2013-07-09

**Authors:** Wolfgang Trapp, Michael Landgrebe, Katharina Hoesl, Stefan Lautenbacher, Bernd Gallhofer, Wilfried Günther, Goeran Hajak

**Affiliations:** 1 Department of Psychiatry, Sozialstiftung Bamberg, St-.Getreu-Straße 14-18, Bamberg, 96049, Germany; 2 Department of Physiological Psychology, Otto-Friedrich University Bamberg, Markusplatz 3, Bamberg, 96045, Germany; 3 Centre for Psychiatry, Justus Liebig University School of Medicine Gießen, Am Steg 22, Gießen, 35392, Germany

**Keywords:** Schizophrenia, Cognition, Cognitive remediation, Outcome, Prediction, Clinical course

## Abstract

**Background:**

Cognitive deficits are stable features of schizophrenia that are linked to functional outcome. Cognitive remediation approaches have been proven successful in ameliorating these deficits, although effect sizes vary considerably. Whether cognitive deficits are serious predictors of clinical outcome is less clear.

**Methods:**

Sixty patients suffering from schizophrenia were included in our sample, thirty of them received computer-assisted cognitive training, and thirty received occupational therapy. For a subsample of 55 patients, who could be traced over a period of five years after the end of the cognitive remediation intervention, time until first relapse and time in psychosis were determined retrospectively from their medical records.

**Results:**

Cognitive remediation significantly improved problem solving, memory and attention with high effect sizes. Employment status, a post test verbal memory performance measure and a measure of executive functioning outperformed all other measures in the prediction of time to relapse, while allocation to treatment group outperformed all other variables in the prediction of both cognitive measures.

**Conclusions:**

Cognitive remediation of neurocognitive deficits thus makes sense in a twofold fashion: It enhances cognition directly and positively acts on clinical course indirectly via improved neurocognition.

**Trial registration:**

German Clinical Trials Register: DRKS00004880

## Background

In the last decades there has been growing interest in the topic of cognitive functioning in schizophrenia, starting from initial hints that cognitive deficits might be significant predictors of social and vocational functioning after (successful) treatment of psychotic symptoms. A huge number of studies were conducted to answer several key questions:

### Do schizophrenia patients suffer from stable cognitive deficits?

A substantial number of studies - as summarized in several meta-analyses [1-3] - report stable deficits in many cognitive domains, such as attention, verbal and visual (working-) memory and executive functions. High effect sizes of 1.0 and above indicate robust impairments. There is empirical evidence that cognitive impairment can be also found before first onset [4] and in persons at risk for schizophrenia (children, siblings, parents of schizophrenia patients) [5,6] while there is currently some debate about whether these cognitive deficits are specific enough to serve as diagnostic criteria for schizophrenia (see for example [7] vs. [8]).

### Are cognitive deficits important?

Many studies have analyzed functional consequences of cognitive deficits and have found small to moderate correlations with functional outcome [9-11]: Cognitive performance seems to be linked to social skills, community functioning, social behavior, social problem solving [12] and even the probability of returning to work or school [13] regardless of potential moderator variables such as age, gender, inpatient status and illness chronicity. Even linkages to further clinical course of illness have been reported by some authors [14-18], although predictive power was limited and there are several studies that could not find any significant relations with neurocognition [19-21].

### Is it possible to remediate neurocognitive deficits?

Three recent meta-analyses considering a huge number of patients have focused on the effects of cognitive remediation in schizophrenia [22-24] reporting effect sizes for overall cognition of 0.38 to 0.45 with effect sizes of about the same for follow-up assessments. Cognitive remediation seems to be more effective when combined with psychiatric rehabilitation interventions and may boost the effects of other remediation programs. The authors agree in their estimation that the effect sizes are rather unaffected by age of the participants, use of computers, frequency and duration of training as well as type of control condition (active or treatment as usual). However, when cognitive remediation therapy is combined with adjunctive psychiatric rehabilitation and when social functioning is considered, strategy based training approaches appear to be superior to pure ‘drill and practice’ approaches [25].

In a former trial [26] we assessed the effects of computer-aided cognitive training using a very game-like and motivating software [27] in forty outpatients suffering from schizophrenia. Twenty of the participants received cognitive training; twenty received occupational therapy twice a week for ten weeks. Enhancing effects on executive functioning level and verbal memory as well as effects on positive and negative symptom levels could be found.

Studies linking cognitive remediation and long-term clinical course of schizophrenia are rare.

The present study describes effects of a cognitive remediation intervention using the same cognitive training software on cognitive performance of 60 schizophrenia inpatients and implications for their further clinical course over a period of 5 years. We hypothesized that our intervention has a strong effect on cognitive performance and that good cognitive performance improves participants’ clinical outcome.

## Methods

### Participants

Sixty inpatients of the psychiatric hospital in Bamberg, Germany were included. All of them fulfilled the International Classification of Diseases-10 (ICD-10), as well as the Diagnostic and Statistical Manual of Mental Disorders-IV (DSM-IV) criteria for schizophrenia and were diagnosed based on the Structured Clinical Interview for the DSM-IV (SCID) [28] performed by physicians blind to treatment allocation. After a complete description of the study, written informed consent was obtained from all subjects. The study adhered to the principles of Good Clinical Practice of the International Conference on Harmonization and the Declaration of Helsinki and was approved by the local ethics board (University of Bamberg).

Thirty patients were included in the experimental group (EG) and thirty patients matched by gender, age and educational level formed the control group (CG). Clinical and demographic characteristics of all participants are described in Table 1.

**Table 1:** Demographic and clinical characteristics

**Characteristic**	**Experimental group (n = 30)**		**Control group (n = 30)**	
	**N**	**%**	**N**	**%**
Gender				
Male	15	50.0	15	50.0
Female	15	50.0	15	50.0
Medication				
Atypical antipsychotic	10	33.3	11	36.7
Typical antipsychotic	1	3.3	4	13.3
Both	19	63.3	15	50.0
Partnership	12	40	8	26.7
Employment status (working)	12	40	13	43.3
Housing in own apartment / house	19	63.3	20	66.7
	Mean	SD	Mean	SD
Age	36.43	12.99	36.87	14.65
Years of education	10.83	3.25	10.47	2.45
Duration of illness	7.93	8.28	8.97	9.65

**Figure 1:**
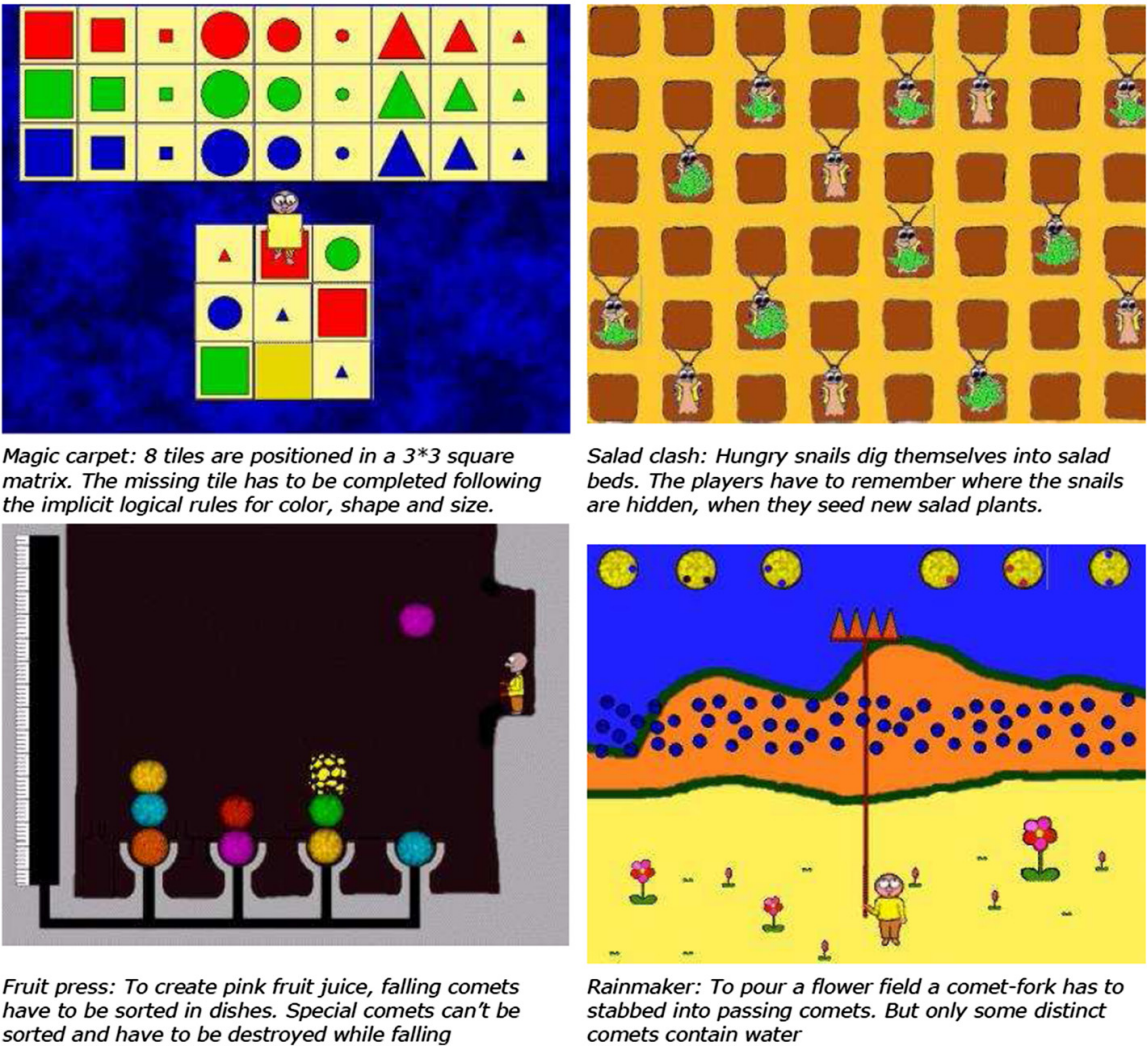
**Screen shots and descriptions of some selected tasks in X**-**Cog.**

The experimental group received four 60-minute training sessions per week for three weeks during their stay at the psychiatric ward (twelve sessions in total) using the “game-like” cognitive training software X-Cog®, that was explicitly designed to motivate patients as much as possible while “playing” the tasks (for more details see [26]). The version administered to the patients consisted of sixteen visuomotor, memory, problem-solving and attention tasks. Participants had to control characters that face several adventurous challenges, such as rescuing a princess which has been captured inside of a maze, protecting salads from hungry snails etc. Each task can be administered in five different levels of difficulty from ‘beginner’ to ‘superprofessional’. Every time a specified level for each task is mastered successfully completed, this is indicated by the software, and the participants then move up to the next level of difficulty. Figure 1 presents screen shots and short descriptions for some selected tasks; a free trial version of the software can be downloaded at http://www.x-cog.de/xcogen.html.

In small groups of three to four patients, 16 tasks that target attention, verbal and visuospatial memory as well as problem solving skills were administered by either one experienced psychologist or occupational therapist who selected the appropriate (and increasing) degree of difficulty based on the participants’ achievement level. All participants completed all 12 training sessions.

Instead of cognitive remediation sessions, CG patients received twelve sessions of occupational therapy (painting and handicraft). Participants were told that they will receive cognitive training or occupational therapy to improve their mental performance.

Additionally to cognitive remediation or occupational therapy, all patients of both groups received antipsychotic drug therapy and periodic visits by a clinical psychiatrist, most of the patients (70 to 80 Percent in both groups) furthermore received sports therapy and music therapy once a week. Furthermore a minority of patients (30 to 40 percent in both groups) attended relaxation therapy groups (twice a week) and received CBT sessions guided by a psychologist (once a week). After their inpatient stay, those patients that could be traced had regular brief (ten to twenty minutes) appointments with their attending psychiatrist about once per month. Both groups did not differ with respect to the frequency of these other therapeutic interventions. Participants were recruited and assigned to either experimental or control group alternatively in two blocks of 15 patients each in order to blind EG participants to the treatment of the CG and vice versa. Symptom levels were obtained before and after treatment for all patients using German versions of the Scale for the Assessment of Positive Symptoms (SAPS) [29], the Scale for the Assessment of Negative Symptoms (SANS) [30] both rated by a senior psychiatrist who was blind to treatment allocation and the Paranoid-Depression Scale (PD-S) self-rating-scale [31]. Inter-rater reliability was not assessed.

Cognitive functioning was assessed using the Wisconsin Card Sorting Test (WCST) [32], the German version of the Wechsler Memory Scale (WMS-R) [33], the Trail Making Test (TMT) Part A und B [34] and two versions of the Continuous Performance Test (CPT) for the assessment of sustained attention: The degraded CPT [35] and the 3–7 CPT [36]: In the degraded version, participants had to respond to a target blurred digit ′3′ in a pseudorandomized sequence of blurred digits, while in the 3–7 version subjects had to respond to each ′7′ that follows a ′3′.

Our sample size provides 85% power to detect an improvement of 30 percent in the WCST ‘total errors’ measure for the experimental group compared to the control group for a two study groups design using a continuous primary endpoint [37] when the estimated standard deviation was taken from the former cognitive remediation study described above.

Five years after end of treatment, time until rehospitalisation (if any) for those patients, who could be traced (28 patients from the EG, 27 patients from the control group) was assessed retrospectively from the medical records of attending physicians / psychiatric hospitals. Each inpatient stay of at least three consecutive days during the follow-up period was considered a rehospitalisation. In fact, the minimum duration of stay found in our sample was 5 days, so every inpatient stay for all of our participants was included in the analyses. Additionally total time in psychosis was obtained as a measure of treatment intensity by adding the days of all inpatient stays during the follow up period.

### Data analysis

In a first step, t-tests for independent samples and chi-square tests were performed to evaluate differences in CG and EG at pre-test time.

Then, to avoid multiple testing, multivariate analyses of variance were computed using treatment group (EG vs. CG) as group factor, “time” (pretest at day 1 vs. posttest at day 14) as repeated measures factor and the following (groups of) dependent variables:

•Attention (degraded and 3–7 CPT measures)

•problem solving (WCST measures and TMT B minus TMT A processing time)

•memory (WMS scores),

•processing speed (TMT A and B processing time),

•positive symptoms (SAPS subscales),

•negative symptoms (SANS subscales) and

•self-rating scores of depressive and paranoid symptoms (PD-S subscales).

For those multivariate analyses that showed significant (p < 0.05) interaction effects ‘group x time’ and thus indicated differential effects of cognitive training, univariate ANOVAS for each dependent variable were performed. Parts of these results for a subset of the neurocognitive measures collected have already been published elsewhere [38].

A Cox regression survival analysis was performed to figure out measures that may predict time until patients’ next relapse. Information about the patient’s clinical and demographical data (partnership, years of education, post-treatment employment status, time since onset of illness and habitation status, medication, gender, age) as well as cognitive achievement level and symptom level at pre and posttest were introduced as independent variables in a stepwise procedure. Based on their standardized regression weights Odds-Ratio values were computed for those variables included in the equation.

To figure out possible predictors of patients’ time in psychosis a linear stepwise regression analysis was performed using time in psychosis as dependent variable and the same set of variables as for the Cox regression described above as predictors.

Since posttest WMS-R verbal memory performance and WCST perseveration errors were the only cognitive variables that significantly predicted time until relapse and time in psychosis, two stepwise linear regression analyses were performed using either verbal memory scores or WCST perseveration errors at posttest as dependent variable in order to search for possible predictors of both cognitive measures. All baseline measures, including “allocation to treatment group” (EG vs. CG) were included as independent variables.

## Results

Clinical and demographic characteristics as well as neurocognitive measures of all participants are described in Tables 1 ,2 and 3 - the groups did not differ in any of the measures shown (t-values between .01 and 1.39, n.s.; *χ*^2^ for medication, housing, partnership and working: 2.32, .73, 1.20, .69, n.s.) except for the total number of WCST errors where EG patients showed a trend towards higher scores (t = 1.99, p = .053).

**Table 2:** **Mean values and results of repeated measures analyses of variance**: **neurocognitive measures**

	**EG (n** = **30)**		**CG (n** = **30)**		**Main effect**		**Main effect**		**Interaction**	
	**Pretest**	**Posttest**	**Pretest**	**Posttest**	**“group”**		**“time”**		**“time x group”**	
**Measure**	**mean (SD)**	**mean (SD)**	**mean (SD)**	**mean (SD)**	**F**_**(4/55) mv.**_	**p**	**F**_**(2/57) mv.**_	**p**	**F**_**(2/57) mv.**_	**p**
					**F**_**(1/58) uv.**_		**F**_**(1/58) uv.**_		**F**_**(1/58) uv.**_	
**Attention (multivariate)**					.**578**	.**680**	**5**.**266**	.**001**	**4**.**606**	.**003**
*Degraded CPT omissions*	*15*.*48* (*14*.*45*)	*10*.*54* (*10*.*99*)	*15*.*44* (*14*.*60*)	*13*.*92* (*12*.*77*)	.*029*	.*866*	*5*.*830*	.*019*	*1*.*639*	.*206*
*Degraded CPT comissions*	*2*.*59* (*2*.*28*)	*1*.*03* (*1*.*49*)	*1*.*85* (*1*.*95*)	*1*.*98* (*3*.*45*)	.*038*	.*847*	*6*.*086*	.*017*	*8*.*561*	.*005*
*3*-*7 CPT omissions*	*26*.*05* (*16*.*24*)	*18*.*70* (*13*.*34*)	*23*.*80* (*14*.*23*)	*24*.*07* (*16*.*85*)	.*205*	.*652*	*3*.*537*	.*065*	*4*.*104*	.*047*
*3*-*7CPT comissions*	*1*.*68* (*1*.*71*)	*0*.*89* (*0*.*92*)	*1*.*87* (*2*.*14*)	*1*.*81* (*2*.*35*)	*1*.*562*	.*216*	*4*.*971*	.*030*	*3*.*725*	.*059*
**Memory (multivariate)**					**1**.**557**	.**220**	**63**.**811**	<.**0005**	**51**.**214**	<.**0005**
*WMS composite score* “*verbal memory*”	*83*.*03* (*18*.*16*)	*103*.*27* (*15*.*74*)	*84*.*23* (*24*.*58*)	*87*.*10* (*21*.*63*)	*2*.*267*	.*138*	*47*.*757*	<.*0005*	*26*.*993*	<.*0005*
*WMS composite score* “*visual memory*”	*77*.*23* (*12*.*62*)	*101*.*03* (*12*.*03*)	*83*.*57* (*16*.*44*)	*83*.*27* (*18*.*73*)	*2*.*597*	.*112*	*48*.*653*	<.*0005*	*51*.*170*	<.*0005*
**Problem solving (multivariate)**					**1**.**694**	.**179**	**7**.**895**	<.**0005**	**20**.**416**	<.**0005**
*WCST* % *total errors*	*28*.*69* (*3*.*84*)	*19*.*94* (*3*.*45*)	*25*.*23* (*8*.*71*)	*27*.*78* (*7*.*90*)	*2*.*188*	.*145*	*17*.*427*	<.*0005*	*57*.*793*	<.*0005*
*WCST* % *failure to maintain set*	*6*.*79* (*3*.*80*)	*3*.*28* (*2*.*12*)	*5*.*66* (*4*.*29*)	*6*.*47* (*4*.*82*)	*1*.*263*	.*266*	*10*.*590*	.*002*	*26*.*918*	<.*0005*
*WCST* % *perseveration errors*	*9*.*11* (*4*.*45*)	*2*.*87* (*2*.*22*)	*7*.*53* (*5*.*42*)	*8*.*74* (*6*.*45*)	*3*.*920*	.*052*	*15*.*192*	<.*0005*	*33*.*180*	<.*0005*
*TMT B*-*A*	*80*,*53* (*48*,*93*)	*53*.*13* (*23*.*65*)	*88*.*13* (*56*.*80*)	*77*.*47* (*49*.*42*)	*2*.*147*	.*148*	*14*.*513*	<.*0005*	*2*.*804*	.*046*
**Speed of processing**					**1**.**120**	.**333**	**11**.**489**	<.**0005**	**2**.**026**	.**141**
*TMT A performance time*	*49*.*43* (*20*.*21*)	*40*.*77* (*16*.*49*)	*49*.*90* (*20*.*27*)	*46*.*20* (*15*.*19*)						
*TMT B performance time*	*129*.*97* (*63*.*70*)	*93*.*90* (*37*.*04*	*138*.*03* (*72*.*96*)	*123*.*67* (*56*.*66*)						

**Table 3:** **Mean values and results of repeated measures analyses of variance**: **symptom measures**

	**EG (n** = **30)**		**CG (n** = **30)**		**Main effect**		**Main effect**		**Interaction**	
	**Pretest**	**Posttest**	**Pretest**	**Posttest**	**“group”**		**“time”**		**“time x group”**	
**Measure**	**mean (SD)**	**mean (SD)**	**mean (SD)**	**mean (SD)**	**F**_**(4/55) mv.**_	**p**	**F**_**(2/57) mv.**_	**p**	**F**_**(2/57) mv.**_	**p**
					**F**_**(1/58) uv.**_		**F**_**(1/58) uv.**_		F_**(1/58) uv.**_	
**SAPS scales (multivariate)**					**1**.**372**	.**256**	**36**.**896**	<.**0005**	**4**.**475**	.**003**
*hallucinations*	*1*.*48* (.*82*)	.*22* (.*50*)	*1*.*72* (*1*.*00*)	.*76* (.*89*)	*4*.*210*	.*045*	*126*.*744*	<.*0005*	*2*.*433*	.*124*
*delusions*	*1*.*85* (.*82*)	.*44* (.*41*)	*1*.*79* (.*93*)	*1*.*15* (.*76*)	*3*.*573*	.*064*	*116*.*570*	<.*0005*	*16*.*464*	<.*0005*
*Bizarre behavior*	.*85* (.*73*)	.*29* (.*39*)	.*61* (.*70*)	.*49* (.*49*)	.*243*	.*624*	*17*.*415*	<.*0005*	*3*.*635*	.*062*
*positive formal thought disorder*	.*98* (*1*.*08*)	.*48* (.*68*)	.*91* (.*82*)	.*52* (.*49*)	.*002*	.*963*	*18*.*452*	<.*0005*	.*282*	.*598*
**SANS scales (multivariate)**					**1**.**611**	.**173**	**8**.**128**	<.**0005**	.**631**	.**677**
*Affective flattening or blunting*	*1*.*43* (*1*.*35*)	.*87* (.*83*)	*1*.*93* (*1*.*44*)	*1*.*25* (*1*.*09*)						
*alogia*	*1*.*45* (.*85*)	.*82* (.*91*)	*1*.*74* (*1*.*21*)	*1*.*38* (*1*.*19*)						
*Avolition* / *apathy*	*1*.*18* (*1*.*37*)	.*76* (*1*.*30*)	*1*.*30* (*1*.*47*)	*1*.*01* (.*98*)						
*Anhedonia* / *asociality*	*1*.*49* (*1*.*26*)	.*58* (.*96*)	*1*.*88* (*1*.*24*)	*1*.*26* (*1*.*09*)						
*attention*	*1*.*13* (*1*.*26*)	.*53* (*1*.*21*)	*1*.*45* (*1*.*27*)	.*75* (.*85*)						
**PD**-**S scales (multivariate)**					**2**.**293**	.**110**	**12**.**812**	<.**0005**	**3**.**405**	.**040**
*Paranoid thinking*	*94*.*06* (*8*.*90*)	*78*.*56* (*22*.*02*)	*95*.*53* (*7*.*12*)	*89*.*65* (*15*.*05*)	*4*.*665*	.*035*	*20*.*519*	<.*0005*	*4*.*160*	.*046*
*Depression*	*85*.*83* (*16*.*97*)	*68*.*93* (*24*.*53*)	*83*.*81* (*28*.*92*)	*79*.*64* (*26*.*19*)	.*587*	.*447*	*13*.*749*	<.*0005*	*5*.*017*	.*029*

Results of multivariate and univariate ANOVAS for neurocognitive measures are shown in Table 2. As can be seen, significant multivariate interaction effects could be found for attention, memory and problem solving measures while no differential changes for EG and CG from pre to posttest could be found for processing speed.

Univariate analyses revealed significant interaction effects for degraded CPT commission, 3–7 CPT omissions as well as all problem solving and memory measures. Inspection of mean values shows an improvement of performance only for EG patients while achievement measures appear unchanged for CG patients.

The corresponding results for symptom measures are displayed in Table 3. Only SAPS and PD-S scales show significant multivariate interaction effects. Univariate analyses point to stronger decreases in SAPS ‘delusions’ as well as in self-rated ‘paranoid’ and ‘depressive’ symptoms for EG patients.

Mean time until first relapse for those participants experiencing any relapse was 526.6 days (SD = 425.8, n = 20) for the experimental and 390.1 days (SD = 433.0, n = 19) for the control group. This difference does not reach statistical significance (t(37) = .99, n.s.).

**Figure 2:**
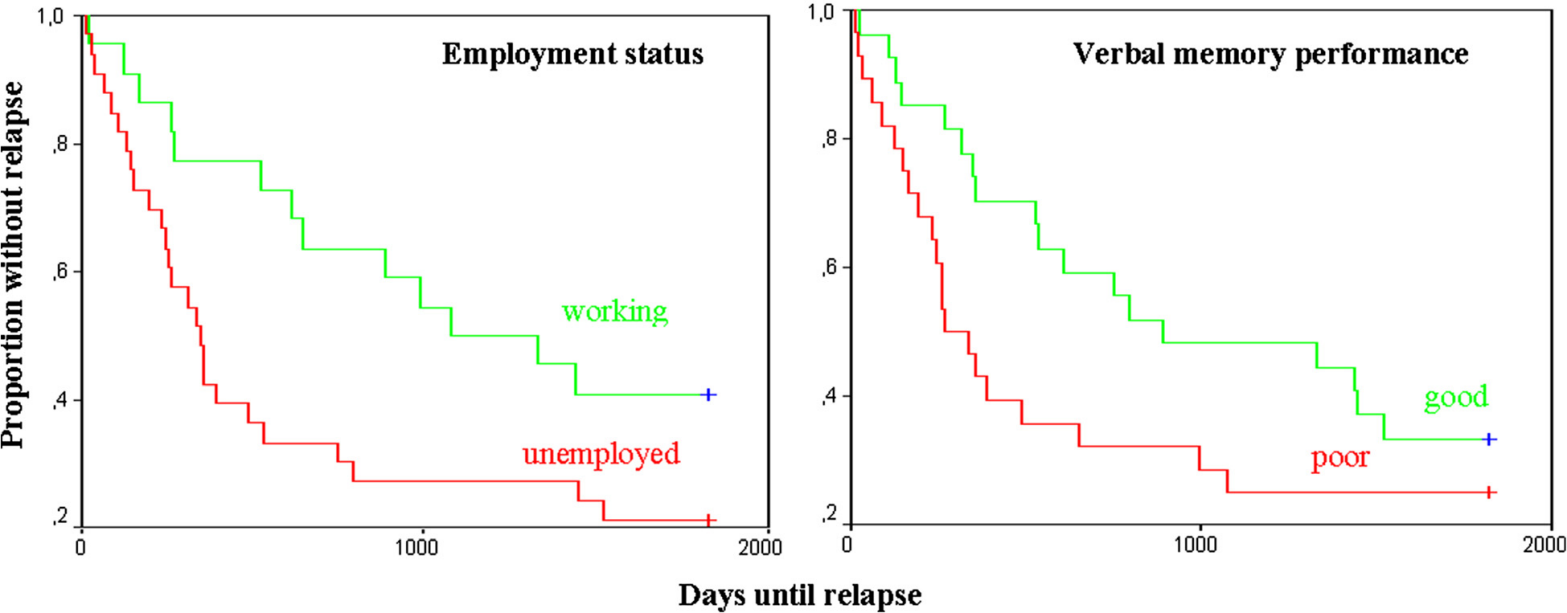
Kaplan Meier survival plots for employment status and verbal memory performance at posttest.

The stepwise procedure in the Cox Regression analysis used to predict time to relapse stopped when two variables – employment status (currently in education or working paid or unpaid in a part- or full-time job vs. unemployed; Wald statistic = 6.54, p = .011, Odds-Ratio = 1/exp(B) = 2.54) and verbal memory score at posttest (Wald statistic = 4.97, p = .026, Odds-Ratio = 1/exp(B) = 2.15) - were included in the equation. Figure 2 shows separate Kaplan Meier survival plots for both variables.

Only three predictors: verbal (beta = .609, p < .0005) and visual memory (beta = .236, p = .007) at baseline as well as “treatment” (beta = .466, p < .0005) remained in the stepwise linear regression procedure’s equation (R^2^ = .739) using post test verbal memory performance as dependent variable.

Average values for time in psychosis for those participants experiencing any relapse was shorter for the cognitive remediation group (mean = 75.0, SD = 51.0) than for the control group (mean = 140.4, SD = 123.6, t(37) = 2.14, p = .043, Cohen’s d = .58). When time in psychosis and number of rehospitalisations is analyzed for the entire sample (with time in psychosis scored 0 for those patients without relapse) differences between both groups do not reach a two-sided α < .05 level (mean values: 53.6(55.0) vs. 100.9(120.5), t(53) = 1.86, p = .071, Cohen’s d = .43).

In contrast to the results of the Cox regression for time until relapse, the stepwise linear regression procedure to predict time in psychosis resulted in an equation containing only one WCST variable for the entire sample (post-intervention perseveration errors, beta = .292, p = .031, R^2^ = .085). Two predictors: WCST total errors (beta = .427, p < .0005) at baseline and “treatment” (beta = .625, p < .0005) remained in the stepwise linear regression procedure’s equation (R^2^ = .409) using post-intervention WCST perseveration errors as dependent variable.

**Figure 3:**
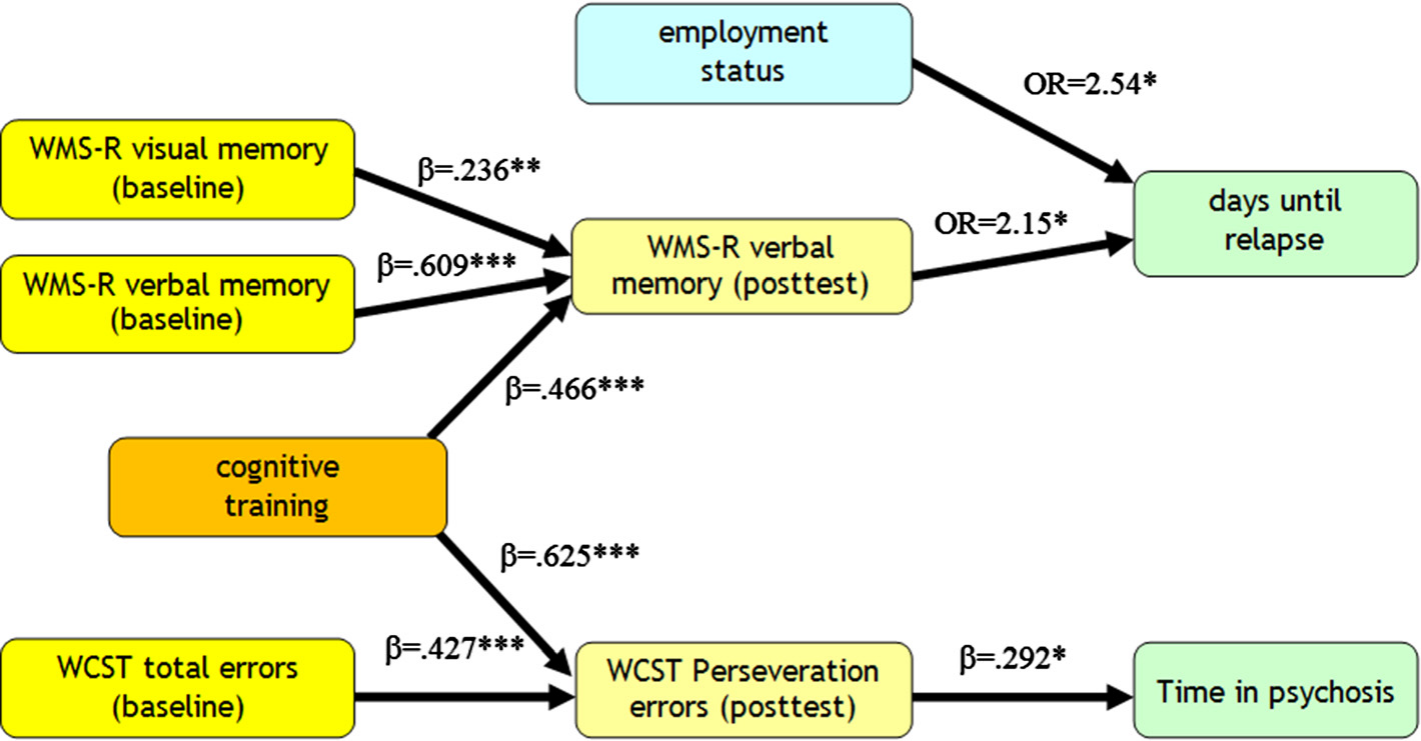
Measures predicting days until relapse and time in psychosis.

Figure 3 summarizes all parameters that directly or indirectly predict time until relapse and time in psychosis in our sample.

## Discussion

This study highlights positive effects of computer-aided cognitive training. We found independent general effects on participants’ memory, attention and executive performance. When time to relapse and time in psychosis five years after the cognitive remediation intervention was analyzed in further detail, verbal memory and problem solving performance at posttest as well as employment status turned out to be the most economic predictors in a stepwise regression procedure. Surely, it is easier to influence cognitive performance during a few weeks inpatient stay and in our sample - even when all other potential influential factors assessed were taken into account – post-intervention cognitive achievement level seems to be solely influenced by performance level before training and training itself.

### Cognitive remediation effect sizes

In our sample effects of CRT on nearly all cognitive domains assessed with effect sizes ranging from medium to high levels (Cohen’s d values range from 0.35 for attention measures to 1.29 for WCST errors, see Table 4) could be found.

**Table 4:** Effect sizes of significantly improved neurocognitive measures

**Measure**	**Cohen’s d**
**Attention**	
*Degraded CPT comissions*	.36
*3*-*7 CPT omissions*	.35
**Memory**	
*WMS composite score* “*verbal memory*”	.85
*WMS composite score* “*visual memory*”	1.13
**Problem solving**	
*WCST* % *total errors*	1.29
*WCST* % *failure to maintain set*	.86
*WCST* % *perseveration errors*	1.22
*TMT B*-*A*	.63

The finding that relatively small effect sizes for attention were obtained corresponds with the results of two recent meta analyses investigating the effects of CRT [22,24]: The authors report similar Cohen’s d values of .28 and .25 for attention as well as lower values of .51 and .57 for problem solving, .37 and .41 for verbal and -.08 and .15 for visual memory compared to our findings. Visuospatial memory was not assessed in our outpatient study for it was not subject to training in contrast to the present contribution. And surprisingly the effect size is very high compared to an overall nonsignificant effect in the collected data of the recent reviews. Unfortunately the authors did not report which cognitive domains were addressed by cognitive remediation, so their findings may be partly due to the fact that nonverbal memory was not subject to cognitive training in many cases.

It is of course difficult to judge why the effect sizes for problem-solving and memory measures are higher in our two samples. One could argue, that the positive effects are just an incidental finding, although they were found in two independent samples comprising 100 patients in total.

One other obvious explanation could be that quality of both studies with respect to allocation to treatment is low (no randomization). But, as could be found recently, independent randomization seems to have no effect on the extent of the effects of cognitive remediation therapy [22].

### Neurocognition and clinical course

As already stated in the ‘introduction’ section, cognitive impairment appears to be a stable feature in schizophrenic patients: It seems to be hardly affected by pharmacological antipsychotic treatment, tends to be stable over time and is present before and at onset of psychosis. Furthermore, there is broad empirical data, that cognitive deficits are linked to functional outcome, and that particular cognitive impairments may even serve as predictors of specific domains of functional outcome [12,39-41]. It is still unclear whether cognitive functioning is related to clinical outcome. By now, only very few studies examined associations of neurocognitive performance to measures like ‘time in psychosis’ or ‘time to first relapse’. Unfortunately, those that discovered significant relationships considered shorter follow-up intervals and, maybe because of this, utilized weaker measures like ‘clinical deterioration’ as criterion for clinical outcome. The probability to get significant correlations of cognitive measures with clinical outcome may of course be higher when they are based on a shorter follow-up period.

Our finding that verbal memory performance and a measure of executive functioning predict time to first relapse and time in psychosis partly matches with results of former studies linking neurocognitive measures to clinical outcome.

Like in our sample, of those studies that revealed significant relationships between cognitive measures and clinical course two [15,18] found significant relationships with verbal memory (although it has to be admitted that in [15] only self rating of memory performance was assessed). Two other studies did not assess verbal memory ([14,16], in [14] verbal learning instead of delayed verbal memory was assessed) and in the remaining contribution [17] TMT-B score, a measure also focusing on cognitive flexibility similar to WCST perseveration errors score outperformed all other measures.

When transition to psychosis is used as a criterion for high-risk samples, verbal memory also repeatedly has been found to be the best predictor [42-44].

There are various possibilities, how cognitive impairment may influence further clinical course: On the one hand, as cognitive impairment is linked to poor functional outcome, insufficient functioning resources may in turn lead to higher stress levels resulting from insufficient coping with life-events and daily hassles. Psychosocial stress is considered a major risk factor for relapse of psychotic symptoms [45-47]. On the other hand good cognitive achievement levels may support treatment compliance and especially may prevent patients from forgetting to take their antipsychotic medication, or from missing appointments with their psychiatrists / consultants [48-50].

Cognitive training itself did not predict time to relapse and only a weak linkage to time in psychosis was found for the entire sample reaching statistical significance only on a one-sided α < .05 level. This is not too surprising because cognitive achievement level of course is not only dependent from cognitive remediation interventions but from other factors like baseline achievement level also. However, in our sample cognitive remediation significantly affected cognitive achievement level even when baseline performance was controlled and therefore may have acted indirectly on further course of illness.

### Limitations and implications for future research

Despite of encouraging results, our sample size of sixty patients is rather small compared to those of other studies examining the effects of CRT, which leads to weaker statistical power and makes it harder to generalize our findings.

Our finding that employment status was only linked to time until rehospitalisation might be due to the fact that only post-intervention employment status was assessed. It is likely that changes in employment level may have occurred and that these changes might have interacted with clinical outcome. Unfortunately we did not collect any data to verify this hypothesis.

Since relapse was determined retrospectively, milder forms of relapse prior to rehospitalisation remained undetected and thus were not included in the analyses.

Unfortunately, our participants were not allocated to treatment in a randomized fashion. This clearly reduces methodological quality and therefore at least the results of the cognitive training intervention should be regarded as preliminary, although, as stated above in this section, current empirical evidence seems to indicate that non-randomized allocation does not influence effect sizes of cognitive training interventions.

## Conclusion

This study provides preliminary evidence that cognitive training may be able to indirectly influence the long-term course of illness in schizophrenic patients: Two cognitive measures and a vocational variable outperformed all other variables as predictors. While it may be difficult to change patients’ employment status during an inpatient stay in a psychiatric hospital, cognitive training could be a powerful instrument to boost cognition and by this means indirectly improve further course of illness.

This is of particular importance considering the chronic and disabling nature of this severe disease starting in early life.

## Competing interests

The authors declare that they have no competing interests.

## Authors’ contributions

WT performed all statistical analyses and drafted the manuscript. BG, WG, WT and GH FG conceived of the study, and participated in its design and coordination. ML and KH helped to draft the manuscript. All authors read and approved the final manuscript.

## Pre-publication history

The pre-publication history for this paper can be accessed here:

http://www.biomedcentral.com/1471-244X/13/184/prepub

## References

[B1] Heinrichs RW Zakzanis KK Neurocognitive deficits in schizophrenia: a quantitative review of the evidence Neuropsychology 1998 12 426 445 9673998 10.1037//0894-4105.12.3.426

[B2] Aleman A Hijman R De Haan EHF Kahn RS Memory impairment in schizophrenia: a meta-analysis Am J Psychiatry 1999 156 1358 1366 10484945 10.1176/ajp.156.9.1358

[B3] Dickinson D Ramsey ME Gold JM Overlooking the obvious: a meta-analytical comparison of digit symbol coding tasks and other cognitive measures in schizophrenia Arch Gen Psychiatry 2007 64 532 542 10.1001/archpsyc.64.5.532 17485605

[B4] Cannon M Caspi A Moffitt TE Evidence for earlychildhood, pan-developmental impairment specific to schizophreniform disorder: results from a longitudinal birth cohort Arch Gen Psychiatry 2002 59 449 456 10.1001/archpsyc.59.5.449 11982449

[B5] Asarnow RF Nuechterlein KH Subotnik KL Fogelson DL Torquato RD Payne DL Neurocognitive impairments in nonpsychotic parents of children with schizophrenia and attentiondeficit/hyperactivity disorder: The University of California, Los Angeles Family Study Arch Gen Psychiatry 2002 59 1053 1060 10.1001/archpsyc.59.11.1053 12418939

[B6] Nuechterlein KH Asarnow RF Subotnik KL Fogelson DL Payne DL Kendler KS The structure of schizotypy: Relationships between neurocognitive and personality disorder features in relatives of schizophrenic patients in the UCLA Family Study Schizophr Res 2002 54 121 130 10.1016/S0920-9964(01)00359-0 11853986

[B7] Bora E Yücel M Pantelis C Cognitive impairment in schizophrenia and affective psychoses: implications for DSM-V criteria and beyond Schizophr Bull 2010 36 1 36 42 10.1093/schbul/sbp094 19776206 PMC2800141

[B8] Keefe RSE Fenton WS How should DSM-V criteria for schizophrenia include cognitive impairment? Schizophr Bull 2007 33 912 920 10.1093/schbul/sbm046 17567627 PMC2632322

[B9] Green MF What are the functional consequences of neurocognitive deficits in schizophrenia? Am J Psychiatry 1996 153 321 330 8610818 10.1176/ajp.153.3.321

[B10] Green MF Kern RS Braff DL Mintz J Neurocognitive deficits and functional outcome in schizophrenia: are we measuring the “right stuff”? Schizophr Bull 2000 26 119 136 10.1093/oxfordjournals.schbul.a033430 10755673

[B11] Green MF Kern RS Heaton RK Longitudinal studies of cognition and functional outcome in schizophrenia: implications for MATRICS Schizophr Res 2004 72 41 51 10.1016/j.schres.2004.09.009 15531406

[B12] Fett AK Viechtbauer W Dominguez MD Penn DL van Os J Krabbendam L The relationship between neurocognition and social cognition with functional outcomes in schizophrenia: a meta-analysis Neurosci Biobehav Rev 2011 35 3 573 588 10.1016/j.neubiorev.2010.07.001 20620163

[B13] Nuechterlein KH Subotnik KL Green MF Ventura J Asarnow RF Gitlin MJ Neurocognitive predictors of work outcome in recent-onset schizophrenia Schizophr Bull 2011 37 Suppl. 2 S33 S40 21860045 10.1093/schbul/sbr084 PMC3160123

[B14] Holthausen EAE Wiersma D Cahn W Kahn RS Dingemans PM Schene AH van den Bosch RJ Predictive value of cognition for different domains of outcome in recent-onset schizophrenia Psychiatry Res 2007 149 71 80 10.1016/j.psychres.2005.07.037 17141329

[B15] Moritz S Krausz M Gottwalz E Lambert M Perro C Ganzer S Naber D Cognitive dysfunction at baseline predicts symptomatic 1-year outcome in first-episode schizophrenics Psychopathology 2000 33 48 51 10.1159/000029119 10601828

[B16] Gråwe RW Levander S Neuropsychological impairments in patients with schizophrenia: stability and prediction of outcome Acta Psychiatr Scand Suppl 2001 408 60 64 11730074 10.1034/j.1600-0447.2001.104s408060.x

[B17] Wölwer W Brinkmeyer J Riesbeck M Freimüller L Klimke A Wagner M Möller HJ Klingberg S Gaebel W German Study Group on First Episode Schizophrenia. Neuropsychological impairments predict the clinical course in schizophrenia Eur Arch Psychiatry Clin Neurosci 2008 258 Suppl 5 28 34 18985291 10.1007/s00406-008-5006-2

[B18] Eberhard J Levander S Lindström E Remission in schizophrenia: analysis in a naturalistic setting Compr Psychiatry 2009 50 3 200 208 10.1016/j.comppsych.2008.08.010 19374962

[B19] Robinson D Woerner MG Alvir JMJ Bilder R Goldman R Geisler S Koreen A Sheitman B Chakos M Mayerhoff D Lieberman JA Predictors of relapse following response form a first episode of schizophrenia or schizoaffective disorder Arch Gen Psychiatry 1999 56 241 247 10.1001/archpsyc.56.3.241 10078501

[B20] Stirling J White C Lewis S Hopkins R Tantam D Huddy A Montague L Neurocognitive function and outcome in first-episode schizophrenia: a 10-year follow-up of an epidemiological cohort Schizophr Res 2003 65 75 86 10.1016/S0920-9964(03)00014-8 14630300

[B21] Buckley PF Harvey PD Bowie CR Loebel A The relationship between symptomatic remission and neuropsychological improvement in schizophrenia patients switched to treatment with ziprasidone Schizophr Res 2007 94 99 106 10.1016/j.schres.2006.12.032 17499480

[B22] Wykes T Huddy V Cellard C McGurk S Czobar P A metaanalysis of cognitive remediation for schizophrenia: methodology and effect sizes Am J Psychiatry 2011 168 472 485 10.1176/appi.ajp.2010.10060855 21406461

[B23] McGurk SR Twamley EW Sitzer DI McHugo GJ Mueser KT A meta-analysis of cognitive remediation in schizophrenia Am J Psychiatry 2007 164 1791 1802 10.1176/appi.ajp.2007.07060906 18056233 PMC3634703

[B24] Grynszpan O Perbal S Pelissolo A Fossati P Jouvent R Dubal S Perez-Diaz F Efficacy and specificity of computer-assisted cognitive remediation in schizophrenia: a meta-analytical study Psychol Med 2011 41 1 163 173 10.1017/S0033291710000607 20380784

[B25] Wykes T Spaulding WD Thinking about the future cognitive remediation therapy–what works and could we do better? Schizophr Bull 2011 37 Suppl 2 S80 S90 10.1093/schbul/sbr064 21860051 PMC3160118

[B26] Trapp W Hasmann A Gallhofer B Schwerdtner J Guenther W Dobmeier M Cognitive Improvement of Schizophrenia Patients: Enhancing Cognition while Enjoying Computer-Aided Cognitive Training Clin Sch Rel Psych 2008 1 4 307 316

[B27] Trapp W X-Cog user manual 2003 Erlangen: Gruner

[B28] First MB Williams JB Spitzer RL Structured Clinical Interview for DSM-IV Axis I Disorders (SCID-I) 1997 Washington, D.C.: American Psychiatric Press, Inc

[B29] Andreasen NC The Scale for the Assessment of Postivie Symptoms (SAPS) Iowa City 1984 IA: The University of Iowa

[B30] Andreasen NC The Scale for the Assessment of Negative Symptoms (SANS) Iowa City 1984 IA: The University of Iowa

[B31] Zerssen D Paranoid-Depressivitäts-Skala sowie Depressivitäts-Skala-Manual 1976 Weinheim: Beltz German

[B32] Young DA Freyslinger MG Scaffolded instruction and the remediation of Wisconsin Card Sorting Test deficits in chronic schizophrenia Schizophr Res 1995 16 3 199 207 10.1016/0920-9964(94)00074-I 7488565

[B33] Wechsler D Wechsler Memory Scale—Revised manual 1987 San Antonio: The Psychological Corporation

[B34] Reitan RM Validity of the trail making test as an indicator of organic brain damage Percept Mot Skills 1958 8 271 276

[B35] Nuechterlein KH Parasuraman R Jiang Q Visual sustained attention: image degradation produces rapid sensitivity decrement over time Science 1983 220 327 329 10.1126/science.6836276 6836276

[B36] Nuechterlein KH Edell WS Norris M Dawson ME Attentional vulnerability indicators, thought disorder, and negative symptoms Schizophr Bull 1986 12 408 426 10.1093/schbul/12.3.408 3764359

[B37] Rosner B Fundamentals of Biostatistics 2011 7 Boston, MA: Brooks/Cole

[B38] Lang U Kognitive Defizite bei Schizophrenien. Evaluation des computergestützten kognitiven Trainings X-Cog 2006 Regensburg: Roderer German

[B39] Bowie CR Leung WW Reichenberg A McClure MM Patterson TL Heaton RK Harvey PD Predicting schizophrenia patients’ real world behavior with specific neuropsychological and functional capacity measures Biol Psychiatry 2008 63 505 511 10.1016/j.biopsych.2007.05.022 17662256 PMC2335305

[B40] Milev P Ho BC Arndt S Andreasen NC Predictive values of neurocognition and negative symptoms on functional outcome in schizophrenia: a longitudinal first-episode study with 7-year follow-up Am J Psychiatry 2005 162 495 506 10.1176/appi.ajp.162.3.495 15741466

[B41] Wittorf A Wiedemann G Buchkremer G Klingberg S Prediction of community outcome in schizophrenia 1 year after discharge from inpatient treatment Eur Arch Psychiatry Clin Neurosci 2008 258 48 58 10.1007/s00406-008-2004-3 17990052

[B42] Brewer WJ Francey SM Wood SJ Jackson HJ Pantelis C Phillips LJ Yung AR Anderson VA McGorry PD Memory impairments identified in people at ultra-high risk for psychosis who later develop first-episode psychosis Am J Psychiatry 2005 162 71 78 10.1176/appi.ajp.162.1.71 15625204

[B43] Lencz T Smith CW McLaughlin D Auther A Nakayama E Hovey L Cornblatt BA Generalized and specific neurocognitive deficits in prodromal schizophrenia Biol Psychiatry 2005 59 863 871 16325151 10.1016/j.biopsych.2005.09.005

[B44] Whyte MC Brett C Harrison LK Byrne M Miller P Lawrie SM Johnstone EC Neuropsychological performance over time in people at high risk of developing schizophrenia and controls Biol Psychiatry 2006 59 730 739 10.1016/j.biopsych.2005.08.028 16388781

[B45] Fowles D Schizophrenia: diathesis-stress revisited Annu Rev Psychol 1992 43 303 336 10.1146/annurev.ps.43.020192.001511 1539945

[B46] Nuechterlein KH Dawson ME Ventura J The vulnerability/ stress model of schizophrenic relapse: a longitudinal study Acta Psychiatr Scand Suppl 1994 382 58 64 8091999 10.1111/j.1600-0447.1994.tb05867.x

[B47] Walker E Diforio D Schizophrenia: a neural diathesis-stress model Psychol Rev 1997 104 667 685 9337628 10.1037/0033-295x.104.4.667

[B48] Lieberman JA Stroup TS McEvoy JP Swartz MS Rosenheck RA Perkins DO Keefe RS Davis SM Davis CE Lebowitz BD Severe J Hsiao JK Clinical Antipsychotic Trials of Intervention Effectiveness (CATIE) Investigators. Effectiveness of antipsychotic drugs in patients with chronic schizophrenia NEngl J Med 2005 13 12 1209 1223 10.1056/NEJMoa051688 16172203

[B49] Kahn RS Fleischhacker WW Boter H Davidson M Vergouwe Y Keet IP Gheorghe MD Rybakowski JK Galderisi S Libiger J Hummer M Dollfus S López-Ibor JJ Hranov LG Gaebel W Peuskens J Lindefors N Riecher-Rössler A Grobbee DE EUFEST study group Effectiveness of antipsychotic drugs in first-episode schizophrenia and schizophreniform disorder: an open randomised clinical trial Lancet 2008 13 9618 1085 1097 18374841 10.1016/S0140-6736(08)60486-9

[B50] Alonso J Croudace T Brown J Gasquet I Knapp MR Suárez D Novick D Health-related quality of life (HRQL) and continuous antipsychotic treatment: 3-year results from the Schizophrenia Health Outcomes (SOHO) study Value Health 2009 12 4 536 543 10.1111/j.1524-4733.2008.00495.x 19900255

